# P-1050. Reduced Urine Cultures and Catheter-Associated Urinary Tract Infection Events with Urine Testing Decision Support

**DOI:** 10.1093/ofid/ofaf695.1245

**Published:** 2026-01-11

**Authors:** William J Lain, Amber Inofuentes, Marissa McKay, Jaswitha Dova, Kristi D Wilkins, Priyanka Kumar, Patrick E H Jackson, Gregory R Madden

**Affiliations:** University of Virginia School of Medicine, Charlottesville, VA; UVA Health, Charlottesville, Virginia; UVA Health, Charlottesville, Virginia; UVA Health, Charlottesville, Virginia; UVA Health, Charlottesville, Virginia; UVA Health University Medical Center, Charlottesville, Virginia; University of Virginia School of Medicine, Charlottesville, VA; University of Virginia, Charlottesville, Virginia

## Abstract

**Background:**

Catheter-associated urinary tract infections (CAUTIs) are among the leading causes of healthcare infections, and excessive urine culturing results in misdiagnosis and inappropriate antibiotic prescriptions. In August 2024, the University of Virginia Health Medical Center implemented a clinical decision support tool to reduce inappropriate urine culture orders and CAUTIs (Figure 1).

**Methods:**

We performed a retrospective quasi-experimental pre-/post-intervention analysis using electronic health record data. Monthly counts of urinalysis with reflex culture, reflex microscopy, and direct culture (without urinalysis for bacteriuria screening in special populations) orders were collected alongside CAUTI events and normalized using the monthly hospital census. Quasi-Poisson regression was used to assess rate changes, incorporating patient-days as an offset variable.

**Results:**

Urinalysis with reflex culture orders declined significantly post-intervention (incidence rate ratio (IRR) 0.47, 95% CI 0.42–0.53, P< 0.001)(Figure 2a), as did direct cultures (IRR 0.76, 95% CI 0.73–0.80, P< 0.001)(Figure 2b). Urinalysis with reflex microscopy orders increased significantly (IRR 3.28, 95% CI 2.82–3.81, P< 0.001)(Figure 2c). Device days decreased significantly (IRR 0.78, 95% CI 0.72–0.84, P < 0.001)(Figure 3a) and sepsis alert firings showed no significant change (IRR: 0.91, 95% CI 0.70–1.18, P=0.472)(Figure 3b). CAUTI events decreased significantly from 1.51 to 0.50 per 10,000 (IRR: 0.31, 95% CI 0.11–0.89, P=0.037)(Figure 4).

**Conclusion:**

A significant reduction in urine culture tests and CAUTIs, along with a significant increase in urine microscopy tests and stable sepsis alerts, suggests that fewer inappropriate cultures were ordered post-intervention. This analysis was limited by time-varying confounders, including a urine retention management tool deployed after the intervention, which likely contributed to reduced catheter use and CAUTIs. These findings support urine testing diagnostic stewardship to improve CAUTI diagnosis and antibiotic stewardship. Further research is needed to investigate granular patient outcomes, inappropriate testing patterns, and antibiotic utilization.

**Disclosures:**

Patrick E H Jackson, MD, Adaptive Phage Therapeutics: Grant/Research Support|Clarametyx, Inc: Grant/Research Support|Pfizer: Grant/Research Support

